# Supplementary material for: Aberrant Epigenetic Regulation in Head and Neck Cancer Due to Distinct EZH2 Overexpression and DNA Hypermethylation
Source: Int J Mol Sci. 2018 Nov 22;19(12):3707. doi: 10.3390/ijms19123707 (PMC6320890; doi:10.3390/ijms19123707)
Supplement: Supplementary file 1 [file ijms-19-03707-s001.pdf]

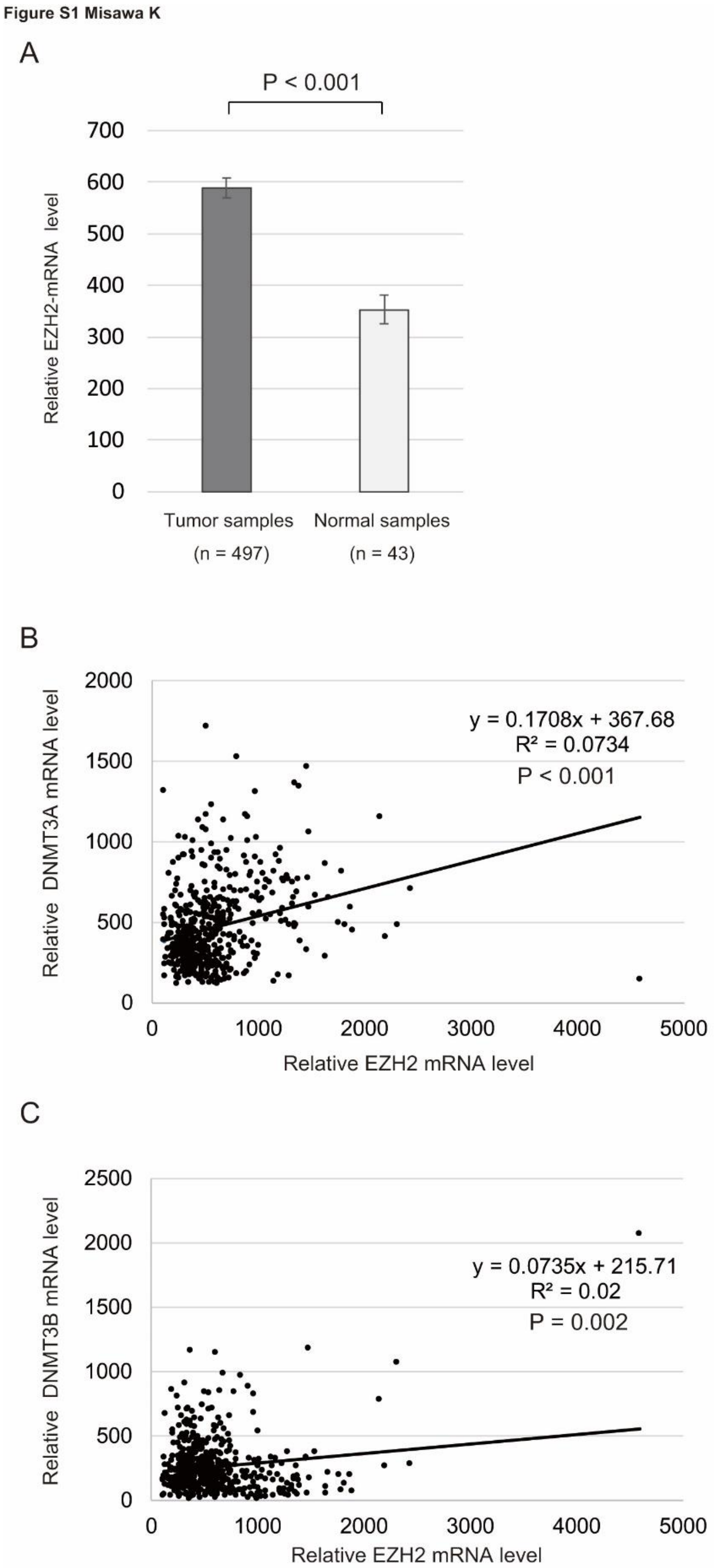

Figure S1. Data regarding EZH2, DNMT3A, and DNMT3B mRNA expression obtained from The Cancer Genome Atlas (TCGA). Data regarding *EZH2*, *DNMT3A*, and *DNMT3B* mRNA expression in head and neck squamous cell carcinoma were obtained from TCGA (https://tcga-data.nci.nih.gov/tcga/) and MethHC (http://methhc.mbc.nctu.edu.tw/php/index.php).

Figure S2 Misawa K

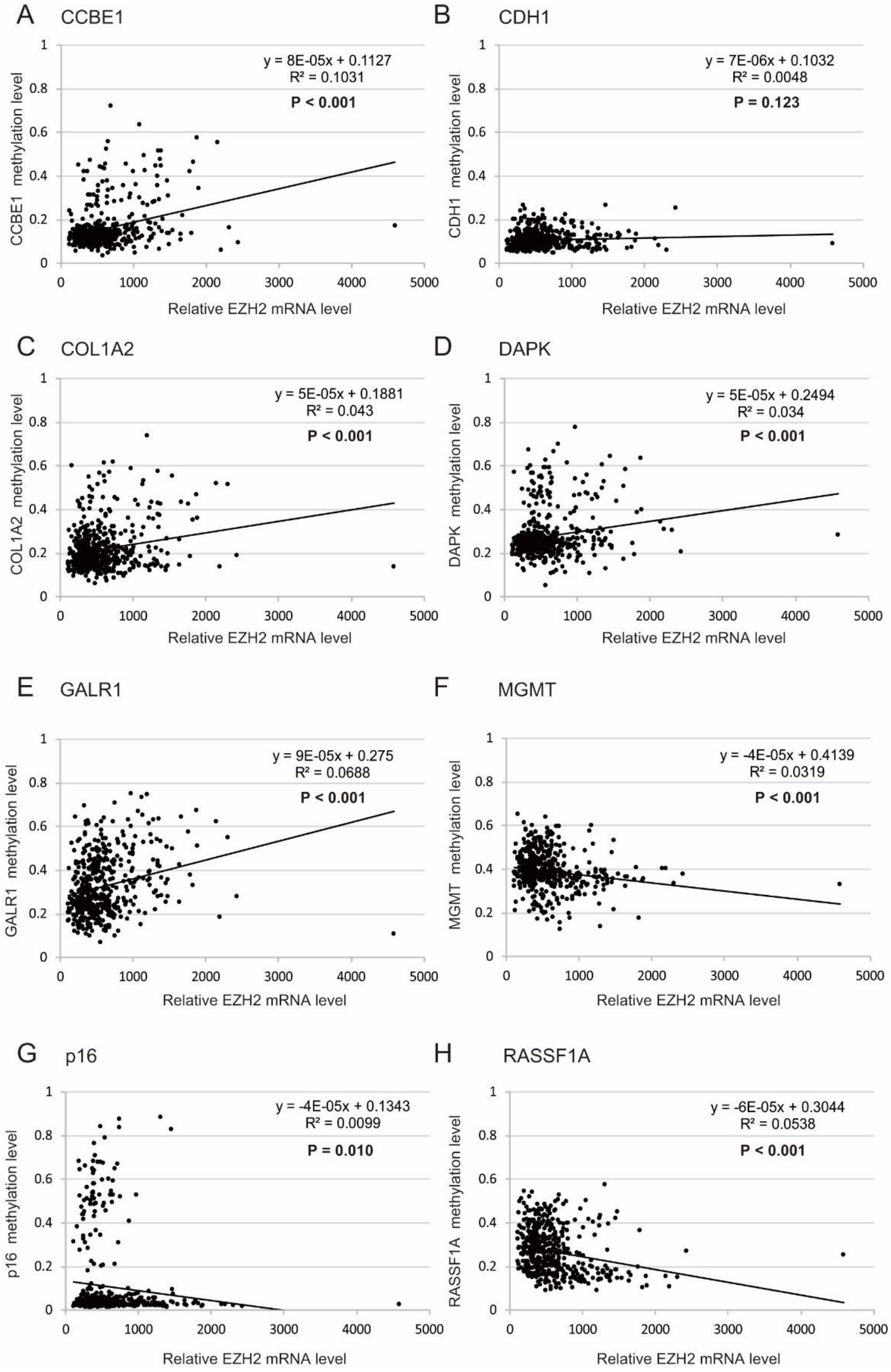

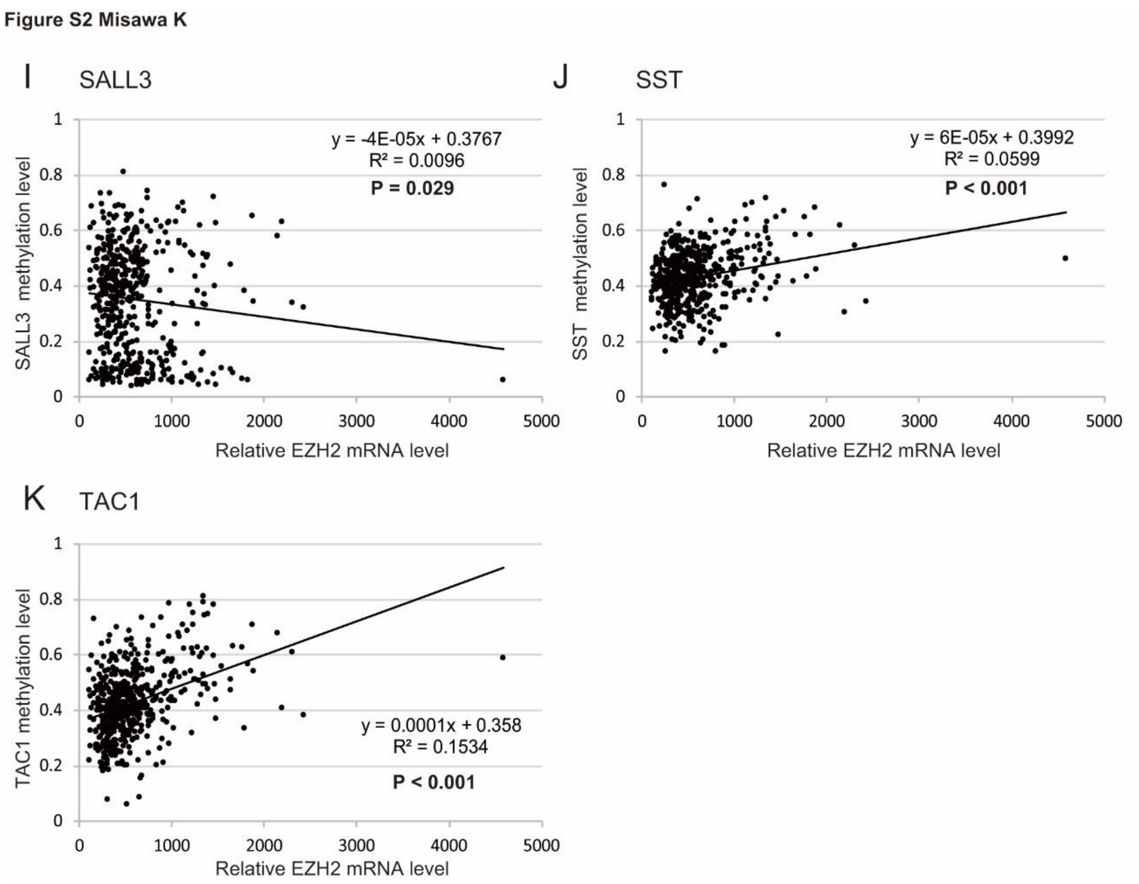

Figure S2. Data regarding DNA methylation obtained from The Cancer Genome Atlas (TCGA).

Table S1. Baseline Characteristics of the 230 Patients.

Patient and tumor characteristics	Original cohort (n = 110)	Validation cohort (n = 120)	Original and Validation cohorts (n = 230)
Age			
Under 70	82 (74.5%)	75 (62.5%)	157 (68.3%)
70 and older	28 (25.5%)	45 (37.5%)	73 (31.7%)
Gender			
Male	87 (79.1%)	103 (85.8%)	190 (82.6%)
Female	23 (20.9%)	17 (14.2%)	40 (17.4%)
Alcohol exposure			
Ever	60 (54.5%)	99 (82.5%)	159 (69.1%)
Never	50 (45.5%)	21 (17.5%)	71 (30.9%)
Smoking status			
Smoker	72 (65.5%)	93 (77.5%)	165 (71.7%)
Non-smoker	38 (34.5%)	27 (22.5%)	65 (28.3%)
Tumor size			
T1	11 (10.0%)	12 (10.0%)	23 (10.0%)
T2	46 (41.8%)	37 (30.8%)	83 (36.1%)
T3	21 (19.1%)	26 (21.7%)	47 (20.4%)
T4	32 (29.1%)	45 (37.5%)	77 (33.5%)

Lympho-node status			
N0	51 (46.4%)	55 (45.8%)	106 (46.1%)
N+	59 (53.6%)	65 (54.2%)	124 (53.9%)
Stage			
I	7 (6.4%)	10 (8.3%)	17 (7.4%)
II	26 (23.6%)	18 (15.0%)	44 (19.1%)
III	19 (17.3%)	24 (20.0%)	43 (18.7%)
IV	58 (52.7%)	68 (56.7%)	126 (54.8%)
Recurrence events			
Positive	56 (50.9%)	40 (33.3%)	96 (41.7%)
Negative	54 (49.1%)	80 (66.7%)	134 (58.3%)

Table S2. Q-RT-PCR and Q-MSP primer list.

PCR	Gene	Forward primer 5’-3’	Reverse primer 5’-3’
Q-RT-PCR	EZH2	CCCTGACCTCTGTCTTACTTGTGGA	ACGTCAGATGGTGCCAGCAATA
Q-RT-PCR	DNMT3A	AGTACGACGACGACGGCTA	CACACTCCACGCAAAAGCAC
Q-RT-PCR	DNMT3B	AGGGAAGACTCGATCCTCGTC	GTGTGTAGCTTAGCAGACTGG
Q-RT-PCR	GAPDH	GCACCGTCAAGGCTGAGAAC	TGGTGAAGACGCCAGTCTCTA
Q-MSP	CCBE1	GTCGCGGAGGAGTAGGACGCTT	CTCGAAAACGACGACACCATC
Q-MSP	CDH1	GTGGGCGGGTCGTTAGTTTC	ACCACAACCAATCAACGCGA
Q-MSP	COL1A2	ACGGTAGTAGGAGGTTTCGG	CGCAAAACCCCTAAATCACCGACG
Q-MSP	DAPK	GGATAGTCGGATCGAGTTAACGTC	CCCTCCCAAACGCCGA
Q-MSP	GALR1	GGTTCGCGGTATTCGGTAGT	GGTTCGCGGTATTCGGTAGT
Q-MSP	MGMT	TTCGACGTTCGTAGGTTTTCGC	GCACTCTTCCGAAAACGAAACG
Q-MSP	p16	GTATTTTTTTCGAGTATTCGTTTACGGC	CAAATCCTCTAAAAAAACCGCGA
Q-MSP	RASSF1A	CGTTCGGTTCGCGTTTGTTAGC	TAACCCGATTAAACCCGTACTTCG
Q-MSP	SALL3	GGGGTTCGAGCGTCGTTAGT	CCGTACTCGAAAACCCCGTC
Q-MSP	SST	GGGGCGTTTTTTAGTTTGACGT	AACAACGATAACTCCGAACCTCG
Q-MSP	TAC1	GGCGGTTAATTAAATATTGAGCAGAAAGTCGC	AAATCCGAACGCGCTCTTTCG
Q-MSP	ACTB	TGGTGATGGAGGAGGTTTAGTAAGT	AACCAATAAAACCTACTCCTCCCTTAA

Table S3. EZH2 expression status with the methylation of other eleven genes.

Genes	Methylation status	High (N = 75)	Low (N = 141)	P-values†
CCBE1	Methylated	51	70	
	Unmethylated	24	71	0.010*
CDH1	Methylated	34	74	
	Unmethylated	41	67	0.322
COL1A2	Methylated	35	45	
	Unmethylated	40	96	1
DAPK	Methylated	47	79	
	Unmethylated	28	62	0.386
GALR1	Methylated	46	68	
	Unmethylated	29	73	0.086
MGMT	Methylated	29	52	
	Unmethylated	46	89	1
p16	Methylated	27	48	
	Unmethylated	48	93	1
RASSF1A	Methylated	10	25	
	Unmethylated	65	116	0.444
SALL3	Methylated	51	72	
	Unmethylated	24	69	0.021*
SST	Methylated	66	116	
	Unmethylated	9	25	0.329
TAC1	Methylated	52	85	
	Unmethylated	23	56	0.235

† Fisher's exact probability test.

* P <0.05.
